# AMYPred-FRL is a novel approach for accurate prediction of amyloid proteins by using feature representation learning

**DOI:** 10.1038/s41598-022-11897-z

**Published:** 2022-05-11

**Authors:** Phasit Charoenkwan, Saeed Ahmed, Chanin Nantasenamat, Julian M. W. Quinn, Mohammad Ali Moni, Pietro Lio’, Watshara Shoombuatong

**Affiliations:** 1 Modern Management and Information Technology, College of Arts, Media and Technology, Chiang Mai University, Chiang Mai, 50200 Thailand; 2 Center of Data Mining and Biomedical Informatics, Faculty of Medical Technology, Mahidol University, Bangkok, 10700 Thailand; 3 Bone Biology Division, Garvan Institute of Medical Research, 384 Victoria Street, Darlinghurst, NSW 2010 Australia; 4 Artificial Intelligence and Digital Health Data Science, School of Health and Rehabilitation Sciences, Faculty of Health and Behavioural Sciences, The University of Queensland, St Lucia, QLD 4072 Australia; 5 Department of Computer Science and Technology, University of Cambridge, Cambridge, CB3 0FD UK

**Keywords:** Computational biology and bioinformatics, Computational models, Machine learning

## Abstract

Amyloid proteins have the ability to form insoluble fibril aggregates that have important pathogenic effects in many tissues. Such amyloidoses are prominently associated with common diseases such as type 2 diabetes, Alzheimer's disease, and Parkinson's disease. There are many types of amyloid proteins, and some proteins that form amyloid aggregates when in a misfolded state. It is difficult to identify such amyloid proteins and their pathogenic properties, but a new and effective approach is by developing effective bioinformatics tools. While several machine learning (ML)-based models for in silico identification of amyloid proteins have been proposed, their predictive performance is limited. In this study, we present AMYPred-FRL, a novel meta-predictor that uses a feature representation learning approach to achieve more accurate amyloid protein identification. AMYPred-FRL combined six well-known ML algorithms (extremely randomized tree, extreme gradient boosting, k-nearest neighbor, logistic regression, random forest, and support vector machine) with ten different sequence-based feature descriptors to generate 60 probabilistic features (PFs), as opposed to state-of-the-art methods developed by a single feature-based approach. A logistic regression recursive feature elimination (LR-RFE) method was used to find the optimal *m* number of 60 PFs in order to improve the predictive performance. Finally, using the meta-predictor approach, the 20 selected PFs were fed into a logistic regression method to create the final hybrid model (AMYPred-FRL). Both cross-validation and independent tests showed that AMYPred-FRL achieved superior predictive performance than its constituent baseline models. In an extensive independent test, AMYPred-FRL outperformed the existing methods by 5.5% and 16.1%, respectively, with accuracy and MCC of 0.873 and 0.710. To expedite high-throughput prediction, a user-friendly web server of AMYPred-FRL is freely available at http://pmlabstack.pythonanywhere.com/AMYPred-FRL. It is anticipated that AMYPred-FRL will be a useful tool in helping researchers to identify new amyloid proteins.

## Introduction

Amyloid proteins (AMYs) can form insoluble aggregates that can accumulate to generate extracellular plaques or intracellular protein inclusions in many organs and tissues, most notably as part of pathological processes. In this aggregated form they have a fibrillary morphology and are primarily composed of β-sheet structures^1^. Among pathological amyloids are those important in the pathogenesis of Alzheimer's disease, where they are seen in central nervous system (CNS) plaques. Such plaques can also form from infectious amyloid prion proteins that cause spongiform encephalopathies and beta-amyloid which, when misfolded, can induce other amyloid proteins to similarly misfold and aggregate. Other pathologies are associated with the accumulation of cleaved normal proteins such as amylin/pancreatic islet amyloid polypeptide (IAPP) which is linked to the development of type 2 diabetes^2,3^. A common method for detecting the presence of amyloid proteins in tissues is a histopathologic examination with the use of histochemical stains such as Congo red and thioflavin T. They can also be detected by mass spectrometry testing to confirm the type and pattern of amyloid deposited^4^. Proteins that form amyloid fibrils are extremely diverse group and lack any sequence or structural homology^5–8^. Identification and characterization of the AMY type present in a tissue of interest is an important step needed to understand such pathological processes and design new therapies, such as small molecules that can inhibit AMY aggregation. Such approaches notably include techniques such as liquid chromatography-tandem mass spectrometry (LC–MS/MS), but while these methods are accurate they are generally costly, technically demanding and time consuming.

Computational tools have previously been developed to study β-amyloid aggregation propensity (β-propensity)^9,10^, amyloidogenicity^11^ and characterize AMY aggregation-prone regions (APRs)^12–15^, the latter being of great importance for the understanding various human pathologies^16^. Several previous studies have shown that physicochemical properties, such as hydrophobicity, β-propensity and buriedness, are important factors for identifying APRs. Recently, Prabakaran et al. developed a new ensemble-based approach called Aggregation Nucleation Prediction in Peptides and Proteins (ANuPP)^16^. In order to overcome the limitations of exiting APR-based predictors as mentioned in Prabakaran et al.^16^, the ANuPP predictor was designed to be a versatile tool able to identify potential APRs in peptides and proteins. ANuPP achieved an area under the operator curve (AUC) of 0.831, as evaluated by tenfold cross-validation test, while this method gave an AUC of 0.883 on a blind test dataset. Their comparative results indicated that ANuPP achieved superior predictive performance than existing methods (AGGRESCAN^9^, Fish Amyloid^17^, GAP^15^, Pasta2^18^, TANGO^13^ and WALTZ^12^). Detailed information for these APRs-based predictors is provided in an article by Prabakaran et al.^19^.

To the best of our knowledge, only a few computational methods, RFAmyloid^9^, iAMY-SCM^10^, PredAmyl-MLP^11^ and Mukhtar et al.’s method^12^ have so far been developed to predict amyloid proteins from a given sequence. In 2018, Niu et al.^20^ proposed the first sequence-based tool named RFAmyloid for discriminating AMYs from non-amyloid proteins (non-AMYs). RFAmyloid was created by combining a random forest (RF) algorithm with various feature encoding methods on a benchmark dataset that included 165 AMYs and 382 non-AMYs. Charoenkwan et al.^21^ developed the iAMY-SCM, a simple and interpretable model based on a scoring card method (SCM) trained with estimated dipeptide propensity scores. According to the findings of Charoenkwan et al.^21^, iAMY-SCM performed at a comparable level to that of RFAmyloid, as evaluated via cross-validation and independent testing. Most recently, Li et al.^22^ and Mukhtar et al.^23^ used amino acid composition (AAC), tripeptide composition (TPC), physicochemical properties of amino acids (AAI), secondary structure-based alignments, and the segmented-position specific scoring matrix (PSSM) method to improve predictive performance. The computational approaches mentioned above each had their own merits and sparked interest in amyloid protein identification research. However, there are a few issues that need to be addressed. Firstly, all of the existing methods were developed by the single feature-based approach that was based on one single ML algorithm. Thus, their predictive performance may not be robust in all cases. Secondly, PredAmyl-MLP^22^ and Mukhtar et al. methods^23^ were developed and evaluated using cross-validation only. As a result, their AMY candidate identification performance is limited in generalizability. Finally, the overall predictive performance of the existing methods is still insufficient for real-world applications.

**Figure 1:**
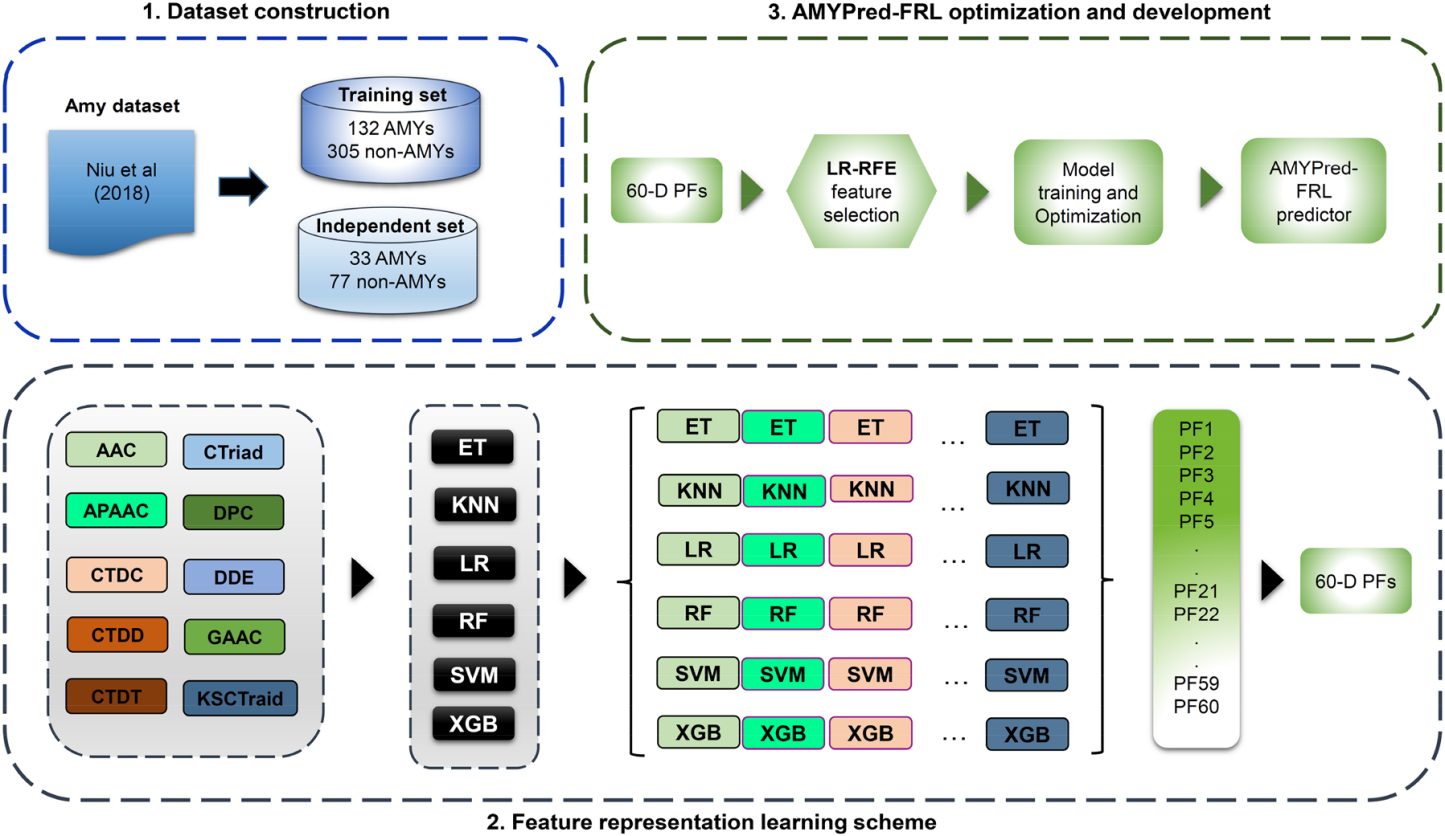
Schematic flowchart of the development of **the** AMYPred-FRL. It consists of dataset construction, feature extraction, baseline models construction and the final meta-based model development.

In this study, we propose a novel machine-learning meta-predictor called the AMYPred-FRL that is designed to further improve the prediction accuracy of amyloid proteins. The overall framework of AMYPred-FRL is shown in Fig. 1. In this predictor, the feature representation learning (FRL) approach was employed to generate 60 probabilistic features (PFs) by combining ten different sequence-based feature descriptors with six well-known ML algorithms. Briefly, the former included AAC, amphiphilic pseudo-amino acid composition (APAAC), composition in CTD (CTDC), conjoint triad (CTriad), dipeptide composition (DPC), dipeptide deviation from the expected mean (DDE), distribution part of CTD (CTDD), grouped amino acid composition (GAAC), k-spaced conjoint triad (KSCTriad), and transition in CTD (CTDT). The latter includes the following ML algorithms: RF, extremely randomized tree (ET), extreme gradient boosting (XGB) k-nearest neighbor (KNN), logistic regression (LR) and support vector machine (SVM). To improve the representation ability of PFs, the feature selection technique was used to determine the best number *m* out of 60 PFs. Finally, selected *m* PFs were used as inputs for training the final meta-predictor using the SVM algorithm (AMYPred-FRL). The independent test revealed that AMYPred-FRL outperformed its constituent baseline models and state-of-the-art methods (RFAmyloid and iAMY-SCM) in terms of accuracy (ACC) of 0.873, sensitivity (Sn) of 0.848, specificity (Sp) of 0.883 and Matthew's Correlation Coefficient (MCC) of 0.710, demonstrating the effectiveness and generalization ability of the method. We believe that our proposed predictor will aid researchers in their efforts to find new and characterize amyloid proteins and enable better drug discovery and development for amyloid proteins that are not well understood.

## Materials and methods

### Dataset preparation

The Amy dataset constructed by Niu et al.^20^ had previously been used to train and develop the four existing state-of-the-art methods (RFAmyloid^20^, iAMY-SCM^21^, PredAmyl-MLP^22^ and Mukhtar et al.’s method^23^). The Amy dataset was used as the benchmark dataset to compare the performance of the proposed method to the four existing state-of-the-art methods. There are 165 AMYs and 382 non-AMYs in the Amy dataset, which are considered as positive and negative samples in this study, respectively. It should be noted that the sequence identity between AMYs and non-AMYs in the Amy dataset exhibited a sequence redundancy of < 50%. In order to test the generalization ability of the proposed method, the Amy dataset was randomly divided into training and independent datasets using the same procedure as the previous two methods (RFAmyloid^20^ and iAMY-SCM^21^). This resulted in training and independent datasets consisting of (132 AMYs and 305 non-AMYs) and (33 AMYs and 77 non-AMYs), respectively.

### Feature extraction

AAC descriptors represent the occurrence frequency of standard amino acids in a protein sequence^24–26^. For the *i*th amino acid, its occurrence frequency (*aa*(*i*)) is represented by:

1 $$ aa\left( i \right) = \frac{{AA_{i} }}{L} $$

where *AA*_*i*_ is the count of occurrences for the *i*th amino acid and *L* is the length of the protein. DPC descriptors represent the occurrence frequency of all possible dipeptides in a protein sequence. For the *i*th dipeptide, its occurrence frequency (*dp*(*i*)) is represented by:

2 $$ dp(i) = \frac{{DP_{i} }}{L - 1} $$

where *DP*_*i*_ is the count of occurrences of the *i*th dipeptide. Final vectors for AAC and DPC are represented as 20- and 400-dimension (20-D and 400-D, respectively) feature vectors, respectively^21,27–29^.

The APAAC descriptor was introduced by Chou^30^ for solving the problem of sequence-order information. The vector for APAAC is represented as a (20 + ${ }2{\uplambda }$)-D feature vector, which is represented by:

3 $$ P = \left[ {\begin{array}{*{20}c} {\begin{array}{*{20}c} {x_{1} } \\ {x_{2} } \\ \end{array} } \\ {\begin{array}{*{20}c} {\begin{array}{*{20}c} \ldots \\ {x_{20} } \\ \end{array} } \\ {\begin{array}{*{20}c} {\begin{array}{*{20}c} {\begin{array}{*{20}c} {x_{20 + 1} } \\ \ldots \\ \end{array} } \\ {x_{20 + \lambda } } \\ \end{array} } \\ {x_{20 + \lambda + 1} } \\ {\begin{array}{*{20}c} {x_{20 + \lambda + 2} } \\ \ldots \\ {x_{20 + 2\lambda } } \\ \end{array} } \\ \end{array} } \\ \end{array} } \\ \end{array} } \right] $$

where the first 20-D feature vector $(x_{1} ,\;x_{2} , \ldots ,\;x_{20} )$ represents the above-mentioned AAC feature descriptor and the remaining 2λ-D feature vector represents the set of correlation factors that reveal physicochemical properties such as hydrophobicity and hydrophilicity in a protein. In this study, parameters of APAAC (the discrete correlation factor λ and weight of the sequence information $\omega$) were estimated by varying $\omega$ and λ values from 0 to 1 and 1 to 10, respectively, with step sizes of 0.1 and 1 as evaluated on the training dataset via the tenfold cross-validation procedure. After performing parameter optimization, $\omega$ and λ values of 0.5 and 10, respectively, were used. The parameter optimization in the current study is the same as employed in our previous studies^31–34^.

Regarding GAAC descriptor, it accounts for properties for all twenty amino acids that can be categorized into five classes including aliphatic group, aromatic group, positive charge group, negatively charged group and uncharged group (Supplementary Table S1). Thus, the vector for GAAC is a 5-D feature vector. The CTD method describes the overall composition of amino acid properties of protein sequences^35^. This method provides three different feature descriptors consisting of the combination (C), transformation (T) and distribution (D)^36^. These three different feature descriptors are based on 13 different physicochemical properties including hydrophobicity, normalized van der Waals volume, polarity, polarization, charge, secondary structure and solvent accessibility^37^. Particularly, CTDC, CTDD and CTDT represent 39-D, 195-D and 39-D feature vectors, respectively. Further details of CTDC, CTDD and CTDT descriptors are described in the work of Xiao et al.^38^.

The CTriad descriptor considers the tripeptide as a single unit for describing protein sequences^39^. All twenty amino acids are classified into seven classes according to their physiochemical properties. As a result, the vector for CTriad is a 343-D feature vector. In the meanwhile, the KSCTriad descriptor is the modified version of CTriad where it provides additional information pertaining to continuous amino acid units as separated by any *k* residues where *k* has a value of 0–5 with an interval of 1^40^. Furthermore, the KSCTriad is a 343-D feature vector. Moreover, the DDE descriptor integrates three property classes including DPC, the theoretical mean (TM) and the theoretical variance (TV)^22^. Particularly, the final vector for DDE is a 400-D feature vector. All these ten sequence-based feature descriptors can be calculated using the iFeature software package^37^.

### Identification of informative features

The extraction of salient features has a crucial influence on the design of computational models. However, taking into account all of the original features may contain irrelevant, redundant, or noisy information that may have a negative impact on the predictive ability of the models. Consequently, capturing significant conserved features is critical in this regard. Here, we used a two-way feature selection approach based on a logistic regression-recursive feature elimination to extract a subset of prominent attributes (LR-RFE). To the best of our knowledge, the LR in conjunction with the recursive feature elimination (RFE) approach is firstly used in AMY identification research. It is a backward iterative process of removing trivial features. The procedure of the LR-RFE method can be described as follows. Firstly, each feature importance is determined using the *L*1-regularized logistic regression (*L*1-LR) method. Specifically, the objective function of the *L*1-LR method for $n$ samples is represented by:

4 $$ \min \left( {\alpha ,\beta } \right)\ell + \lambda \left| \beta \right|_{1} = \frac{1}{n}\mathop \sum \limits_{i = 1}^{n} {\text{log}}(1 + {\text{exp}}( - (\beta^{T} a_{i} + \alpha_{i} y_{i} ))) + \lambda \mathop \sum \limits_{i = 1}^{n} \left| {\beta_{i} } \right|{ } $$

where $\beta_{i}$ represents the predictive ability of the *i*th feature. In the meanwhile, $a_{i}$ represents $x_{i} y_{i}$ and *L*1 norm $\left| \beta \right|_{1}$ represents $\sum\nolimits_{i = 1}^{n} {\left| {\beta_{i} } \right|}$ where λ > 0. Features exhibiting the largest value of $\beta_{i}$ are retained while features with the lowest values of $\beta_{i}$ are discarded from the attribute set. Secondly, features are ranked followed by sorting in descending order according to $\beta_{i}$. The LR-RFE method repeats this process for *N* times until an optimal feature set with higher prediction performance is obtained.

### Feature representation learning framework

Unlike traditional feature encodings, the FRL method employs a wide range of feature descriptors to provide sufficient information from various perspectives. The FRL method, originally proposed by Wei et al.^41^, has recently been shown to perform well in identifying various functional activities of peptides^41–44^. Inspired by the original FRL method^41^, we developed and implemented the extended version of the FRL method by combining it with various ML classifiers^34,41–43,45,46^. The used FRL method and the AMPred-FRL development are described further below.

#### Baseline models generation

As summarized in Table 1, we employed ten different feature encodings (AAC, APAAC, CTDC, CTDD, CTDT, CTriad, DPC, DDE, GAAC, and KSCTriad) as derived from three different properties (composition information, composition–transition–distribution information and physicochemical properties). Subsequently, each feature descriptor was individually employed for training baseline models using six different ML algorithms (ET, KNN, LR, RF, SVM and XGB). In total, 60 baseline models (6 MLs × 10 encodings) were created using the Scikit-learn package in Python with default parameters (version 0.22)^47^. The procedure for building baseline models was performed in a similar fashion to the one used in our previous studies^34,45,46,48^.

**Table 1:** Summary of ten different feature encodings along with their corresponding description and dimension.

Order	Descriptors	Description	Dimension	References
1	AAC	Amino acid composition	20	^56^
2	APAAC	Amphiphilic pseudo-amino acid composition	22	^30^
3	CTDC	Percentage of particular amino acid property groups	39	^35,36,57^
4	CTDD	Distribution of amino acid properties in sequences	195	^2–4^
5	CTDT	Percentage of mutual conversion in amino acid properties	39	^35,36,57^
6	CTriad	Conjoint triad	343	^39^
7	DDE	Dipeptide deviation from expected mean	400	^58,59^
8	DPC	Dipeptide composition	400	^35,58^
9	GAAC	Grouped amino acid composition	5	^37,56,60^
10	KSCTriad	K-spaced conjoint triad	343	^37^

#### Feature representation generation

Each baseline model can provide two types of information including probabilistic information and class information. For a given protein sequence *P*, its probabilistic information was obtained from the predicted probability. In the case of the class information, if the predicted probability of *P* exceeds 0.5, the protein sequence belongs to AMY, otherwise, the protein sequence belongs to the non-AMY class. Subsequently, we concatenated all of the predicted probability and predicted class as derived from 60 baseline models in order to obtain two 60-D feature vectors, which are referred to as probabilistic feature (PF) and class feature (CF) vectors, respectively. In the meanwhile, the combination of PF and CF is referred to as PCFs that essentially represents a 120-D feature vector. The PF and CF are represented by:

5 $$ {\text{PF}} = \left[ {P({\text{M}}_{1} , \;F_{1} } \right), \ldots ,\;P({\text{M}}_{i} , \;F_{j} ), \ldots ,\;P({\text{M}}_{s} , \;F_{t} ))]^{T} $$

6 $$ {\text{CF}} = \left[ {C({\text{M}}_{1} , \;F_{1} } \right), \ldots ,\;C({\text{M}}_{i} , \;F_{j} ), \ldots ,\;C({\text{M}}_{s} , \;F_{t} ))]^{ T} $$

where $P({\text{M}}_{i} , \;F_{j} )$ and $C({\text{M}}_{i} , \;F_{j} )$ were obtained using the *i*th baseline model with the *j*th feature descriptor. The PF, CF and PCF are considered as new feature vectors.

#### Feature representation optimization

The optimal feature sets of PF, CF, and PCF were determined using the LR-RFE method so as to improve the feature representation ability. There are three main steps for determining the optimal feature vectors using the LR-RFE method, which are as follows: (i) 60 PFs, 60 CFs and 120 PCFs were ranked using the *L*1-LR method, (ii) the RFE algorithm was applied for selecting optimal features using an interval of 5 that finally led to the selection of 20 PFs, 30 CFs and 10 PCFs, (iii) all feature subsets were used to train LR models individually that are then used for developing the meta-predictor. The feature subset with the highest cross-validation ACC was considered as the optimal feature set and used for the meta-predictor development.

#### AMPred-FRL development

In this study, the FRL method systematically uses these baseline models to build a single hybrid model. After obtaining the best feature sets, they were individually fed into the LR algorithm (referred herein as mLR) to produce the final meta-predictor. To improve the predictive performance even further, parameters for each of the three mLR models were estimated using the tenfold cross-validation procedure (i.e. the search range is presented in Supplementary Table S2).

### Performance evaluation metrics

The predictive performance of our proposed model, baseline models and the two state-of-the-art methods is evaluated and compared using five common performance measures as follows: ACC, sensitivity (Sn), specificity (Sp), Matthew's Correlation Coefficient (MCC) and area under the receiver-operating curves (AUC)^46,49^. These performance measures are described by the following equations:

7 $$ {\text{ACC}} = \frac{{{\text{TP}} + {\text{TN}}}}{{\left( {{\text{TP}} + {\text{TN}} + {\text{FP}} + {\text{FN}}} \right)}} $$

8 $$ {\text{Sn}} = \frac{{{\text{TP}}}}{{\left( {{\text{TP}} + {\text{FN}}} \right)}} $$

9 $$ {\text{Sp}} = \frac{{{\text{TN}}}}{{\left( {{\text{TN}} + {\text{FP}}} \right)}} $$

10 $$ {\text{MCC}} = \frac{{{\text{TP}} \times {\text{TN}} - {\text{FP}} \times {\text{FN}}}}{{\sqrt {\left( {{\text{TP}} + {\text{FP}}} \right)\left( {{\text{TP}} + {\text{FN}}} \right)\left( {{\text{TN}} + {\text{FP}}} \right)\left( {{\text{TN}} + {\text{FN}}} \right)} }} $$

where TP, TN, FP and FN represent the number of true positives, true negatives, false positives and false negatives, respectively^50–52^.

## Results and discussion

### Performance evaluation of different baseline models

**Figure 2:**
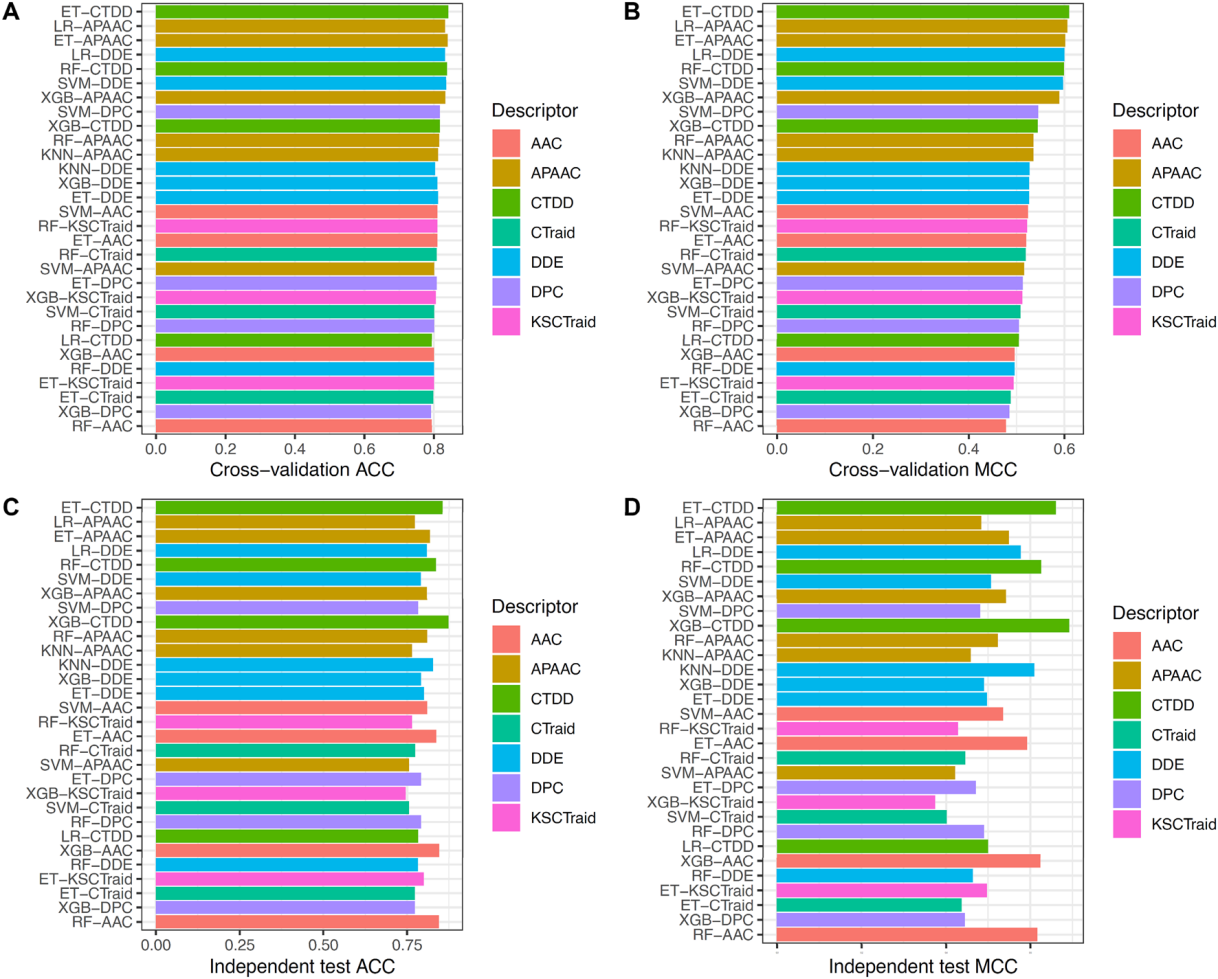
Performance evaluations of top 30 baseline models. (**A**,**B**) Cross-validation ACC and MCC as well as (**C**,**D**) Independent test ACC and MCC.

In this section, we investigated and evaluated the predictive performance of different baseline models trained using ten different feature encodings (AAC, APAAC, CTDC, CTDD, CTDT, CTriad, DPC, DDE, GAAC, and KSCTriatd) and six different ML algorithms (ET, KNN, LR, RF, SVM and XGB) by performing both tenfold cross-validation and independent tests. Their cross-validation and independent test results are provided and visualized in Supplementary Tables S3, S4 and Fig. 2, respectively. As can be seen from Supplementary Table S3, AAC, APAAC, CTDC, CTDD, CTDT, CTraid, DDE, DPC, GAAC and KSCTraid display average cross-validation results for the 2 performance metrics (ACC, MCC) of (0.778, 0.396), (0.822, 0.564), (0.734, 0.281), (0.808, 0.519), (0.745, 0.300), (0.786, 0.455), (0.751, 0.320), (0.756, 0.342), (0.712, 0.209) and (0.787, 0.461), respectively, as obtained from six different ML algorithms. From amongst these ten different feature encodings, it was noted that there were four beneficial feature descriptors for AMY identification consisting of APAAC, CTDD, KCTraid and CTraid that were able to achieve an average MCC value larger than 0.4. Particularly, APAAC, CTDD, KCTraid and CTraid feature descriptors were used as input for the development of LR, ET, RF and RF classifiers that were found to produce the highest cross-validation results, as evaluated by the two performance metrics (ACC, MCC) of (0.833, 0.606), (0.842, 0.610), (0.808, 0.519) and (0.810, 0.522), respectively.

We also investigated the predictive performance of 60 baseline models so as to determine the best performer of these for AMY identification. From Fig. 2 and Supplementary Tables S3, S4, several important observations can be summarized as follows. Firstly, the ten baseline models ranking highest for cross-validation MCC were the following: ET-CTDD, LR-APAAC, ET-APAAC, LR-DDE, RF-CTDD, SVM-DDE, XGB-APAAC, SVM-DPC, XGB-CTDD and RF-APAAC. It was notable that seven out of ten top-ranked baseline models were developed from APAAC and CTDD, which again confirms their importance in AMY identification. Secondly, six out of ten top-ranking baseline models were developed using tree-based ensemble algorithms (RF, ET and XGB). From amongst the ten top-ranking baseline models, RF-based, ET-based and XGB-based classifiers achieved favorable ACC in the range of 0.815–0.842 while LR-based classifiers were found to achieve an ACC of 0.833, which was comparable to these tree-based classifiers. Thirdly, ET-CTDD was found to be the best baseline model as obtained from cross-validation and independent performance (ACC, MCC) of (0.842, 0.610) and (0.855, 0.660), respectively.

### Comparison of class, probabilistic and combined information

In this section, we compared the predictive performance of mLR models as trained with CF, PF and PCF feature vectors. Their cross-validation and independent test results are recorded in Tables 2 and 3, respectively. As can be seen in Table 2, the PF vector could outperform both CF and PCF vectors, which correspondingly achieved the highest values for ACC (0.870), Sn (0.735), MCC (0.687) and AUC (0.912) on the training dataset. In the case of independent test results, we observed that the overall performance of the PF vector was consistently better than those of CF and PCF vectors as indicated by all five performance metrics (i.e., ACC, Sn, Sp, MCC and AUC).

**Table 2:** Cross-validation results for different feature representations using class and probabilistic information.

Features	Dimension	ACC	Sn	Sp	MCC	AUC
CF	60	0.851	0.673	0.928	0.634	0.883
PF	60	0.870	0.735	0.928	0.687	0.912
PCF	120	0.858	0.711	0.921	0.654	0.889
Optimal CF	30	0.883	0.749	0.941	0.717	0.895
Optimal PF	20	0.892	0.780	0.941	0.743	0.925
Optimal PCF	10	0.881	0.765	0.931	0.714	0.919

**Table 3:** Independent test results for different feature representations using class and probabilistic information.

Features	Dimension	ACC	Sn	Sp	MCC	AUC
CF	60	0.836	0.727	0.883	0.610	0.896
PF	60	0.882	0.848	0.896	0.727	0.921
PCF	120	0.855	0.818	0.870	0.668	0.912
Optimal CF	30	0.864	0.879	0.857	0.701	0.914
Optimal PF	20	0.873	0.848	0.883	0.710	0.902
Optimal PCF	10	0.836	0.758	0.870	0.618	0.889

To enhance the predictive performance of mLR models, the LR-RFE method was used for identifying the optimal feature sets of PF, CF and PCF vectors. For the CF, PF and PCF feature vectors, Table 2 shows that when the feature number was set to 30, 20 and 10, respectively, their predictive models could achieve maximal cross-validation performance (ACC and MCC) of (0.867, 0.677), (0.892, 0.743) and (0.881, 0.717), respectively. For the convenience of discussion, the optimal feature vectors of CF, PF and PCF were referred as optimal CF, optimal PF and optimal PCF, respectively. The overall cross-validation performance of the optimal PF was better than that of the optimal CF and the optimal PCF in terms of ACC, Sn, MCC and AUC. In the case of independent test results, the optimal PF outperformed those of the optimal CF and optimal PCF as indicated by three out of five performance metrics (i.e., ACC, Sp and MCC). In particular, the optimal PF achieved an ACC of 0.873, an Sp of 0.883 and an MCC of 0.710 (Table 3). For convenience, the mLR model trained with the 20-D optimal PF will be considered as the final meta-predictor that is herein referred to as the AMYPred-FRL. Details of the optimal feature vectors of CF, PF and PCF are provided in Supplementary Table S5.

### Contribution of new feature representations

**Figure 3:**
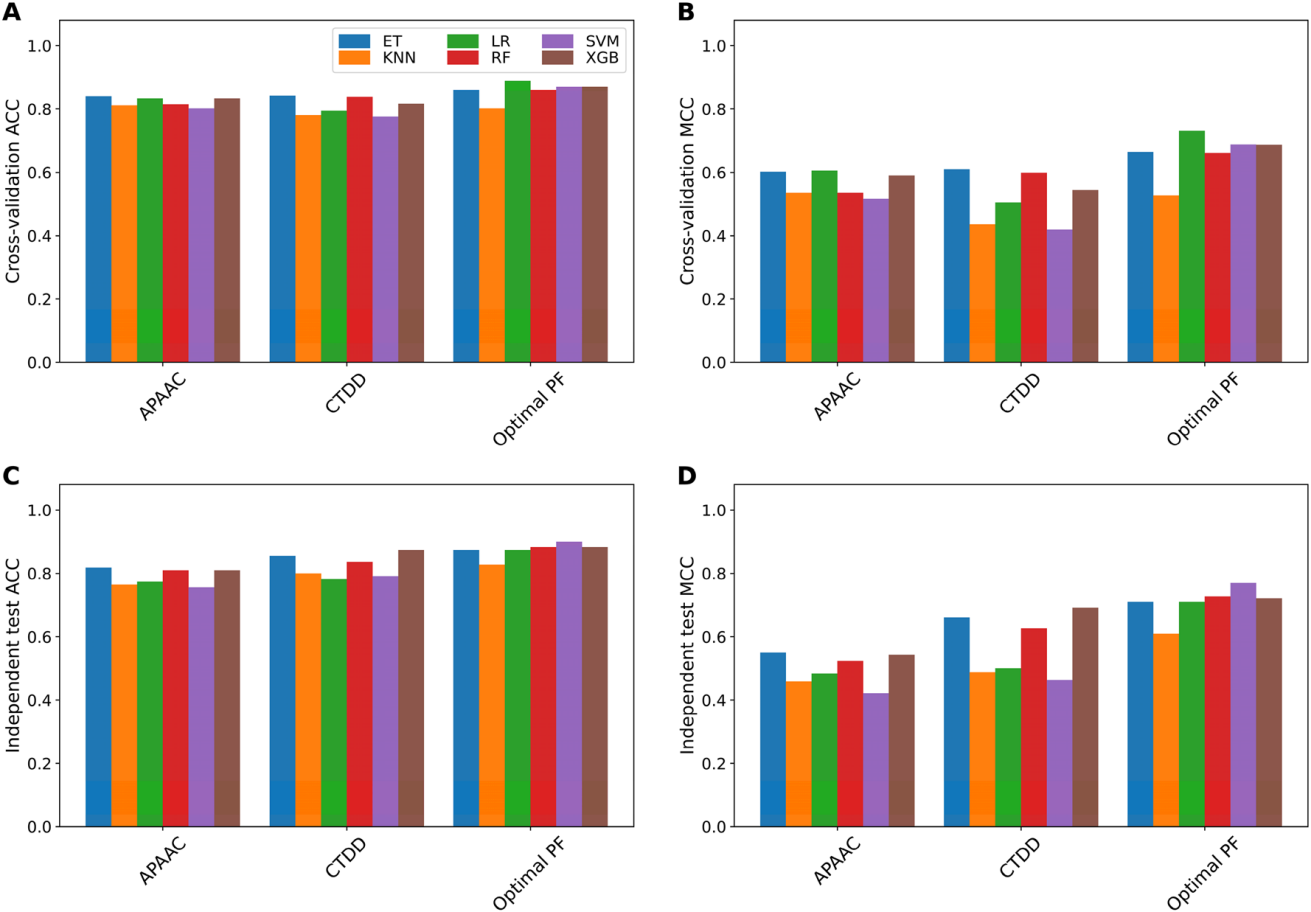
Performance comparison of optimal PF and conventional feature descriptors for six ML algorithms (ET, KNN, LR, RF, SVM and XGB) in terms of cross-validation ACC and MCC (**A**,**B**) and independent test ACC and MCC (**C**,**D**).

**Figure 4:**
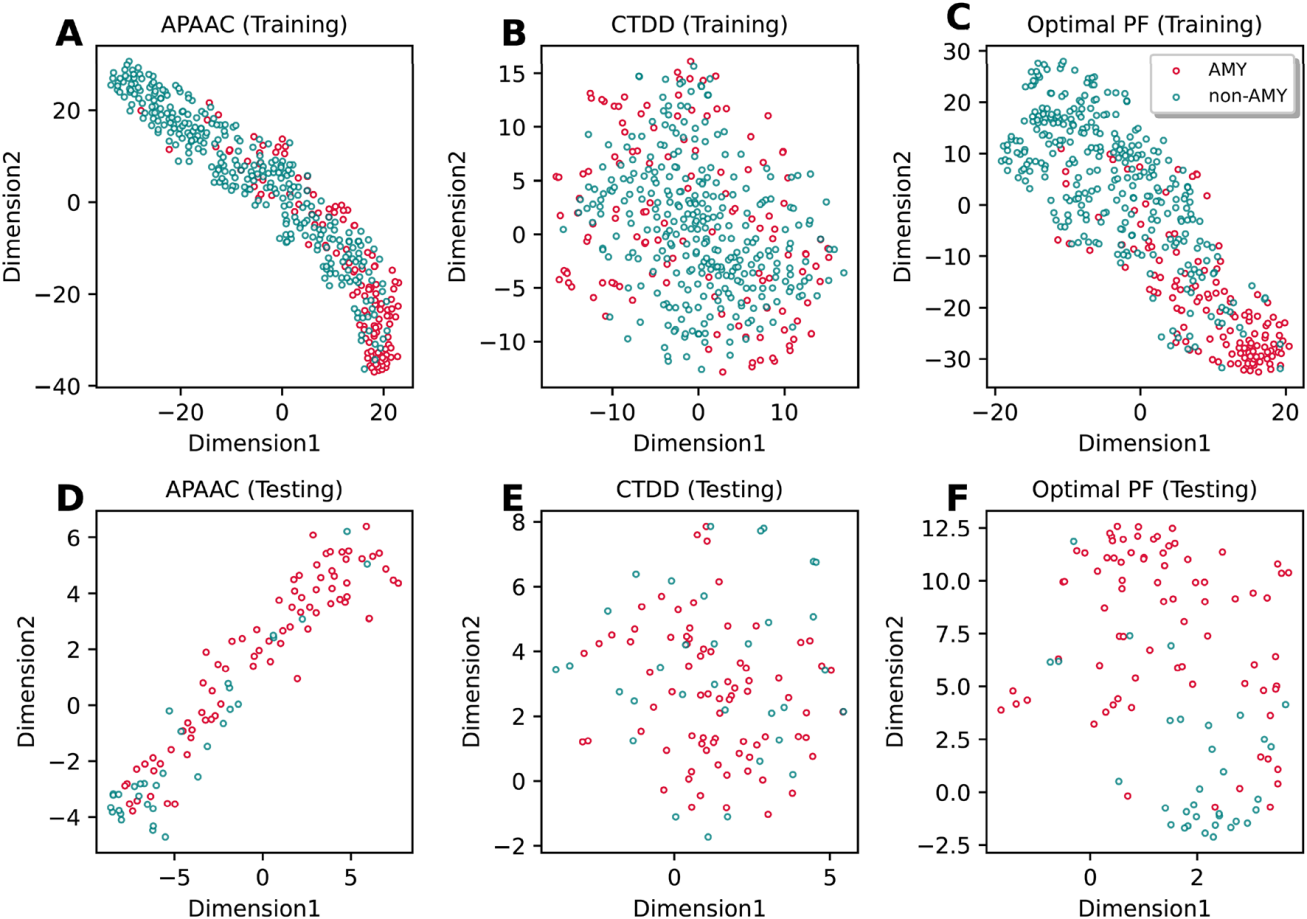
*t-*distributed stochastic neighbor embedding (*t*-SNE) distribution of positive and negative samples on training (**A**–**C**) and independent test (**D**–**F**) datasets. APAAC (**A**,**D**), CTDD (**B**,**E**) and Optimal PF (**C**,**F**).

This section investigates whether the feature representation (i.e., the optimal PF) proposed herein as derived using the FRL approach could improve the prediction accuracy of amyloid protein identification. To demonstrate this point, we compared the performance of the optimal PF and conventional feature descriptors as evaluated by six ML algorithms via cross-validation and independent tests. The feature descriptor with the highest cross-validation MCC was considered to be the optimal descriptor and was used for this comparative analysis. As can be seen from Supplementary Tables S3, S4, optimal descriptors for ET, KNN, LR, RF, SVM and XGB are CTDD, APAAC, APAAC, CTDD, DDE and APAAC, respectively. Comparative results are summarized in Tables 4, 5 as well as Fig. 3. As shown in Table 4, the optimal PF exhibited better performance than those of compared feature descriptors with the exception of KNN. As shown in Table 4, the optimal PF trained with ET, LR, RF, SVM and XGB could achieve a cross-validation MCC of 0.860, 0.888, 0.860, 0.870 and 0.870, respectively, with improvements of 1.8%, 5.5%, 2.20%, 3.5% and 3.7%, respectively. In the case of independent test results, it is observed that the optimal PF vectors could achieve better performance in terms of ACC, Sn and MCC. (Table 5). Secondly, to elucidate the effectiveness of our feature representations, *t*-distributed stochastic neighbor embedding (*t*-SNE) was used to visualize the feature space between our feature representation and the best feature descriptors (i.e., APAAC and CTDD) using the training and independent test datasets. Figure 4 depicts the distribution of the feature space in a 2D representation whereby AMYs (red spots) and non-AMYs (green spots) are shown. As can be noticed in Fig. 4, red and green spots when superimposed with feature descriptors (Fig. 4A,B and D,E) there appear to be overlaps. On the other hand, a clear distinction between red and green spots could be obtained from this feature representation (Fig. 4C,F). This confirmed that the FRL approach could effectively take advantage of variant models for capturing discriminative patterns between AMYs and non-AMYs thereby leading to more accurate AMY identification.

**Table 4:** Performance comparison of new feature representation with conventional sequence-based feature descriptors as evaluated on the cross-validation test.

Method	Feature	ACC	Sn	Sp	MCC	AUC
ET	Optimal PF	0.860	0.719	0.921	0.664	0.915
	CTDD	0.842	0.636	0.931	0.610	0.892
KNN	Optimal PF	0.801	0.665	0.859	0.527	0.762
	APAAC	0.812	0.591	0.908	0.535	0.848
LR	Optimal PF	0.888	0.765	0.941	0.731	0.926
	APAAC	0.833	0.735	0.875	0.606	0.878
RF	Optimal PF	0.860	0.718	0.921	0.661	0.913
	CTDD	0.838	0.636	0.925	0.599	0.884
SVM	Optimal PF	0.870	0.727	0.931	0.687	0.892
	DDE	0.835	0.659	0.911	0.597	0.890
XGB	Optimal PF	0.870	0.726	0.931	0.686	0.917
	APAAC	0.833	0.644	0.915	0.589	0.881

**Table 5:** Performance comparison of new feature representation with conventional sequence-based feature descriptors as evaluated on the independent test.

Method	Feature	ACC	Sn	Sp	MCC	AUC
ET	Optimal PF	0.873	0.848	0.883	0.710	0.925
	CTDD	0.855	0.788	0.883	0.660	0.905
KNN	Optimal PF	0.827	0.788	0.844	0.609	0.816
	APAAC	0.764	0.667	0.805	0.458	0.829
LR	Optimal PF	0.873	0.848	0.883	0.710	0.902
	APAAC	0.773	0.697	0.805	0.484	0.832
RF	Optimal PF	0.882	0.848	0.896	0.727	0.925
	CTDD	0.836	0.788	0.857	0.626	0.897
SVM	Optimal PF	0.900	0.879	0.909	0.769	0.900
	DDE	0.791	0.667	0.844	0.507	0.867
XGB	Optimal PF	0.882	0.818	0.909	0.721	0.924
	APAAC	0.809	0.667	0.870	0.542	0.876

### Mechanistic interpretation of AMYPred-FRL

**Figure 5:**
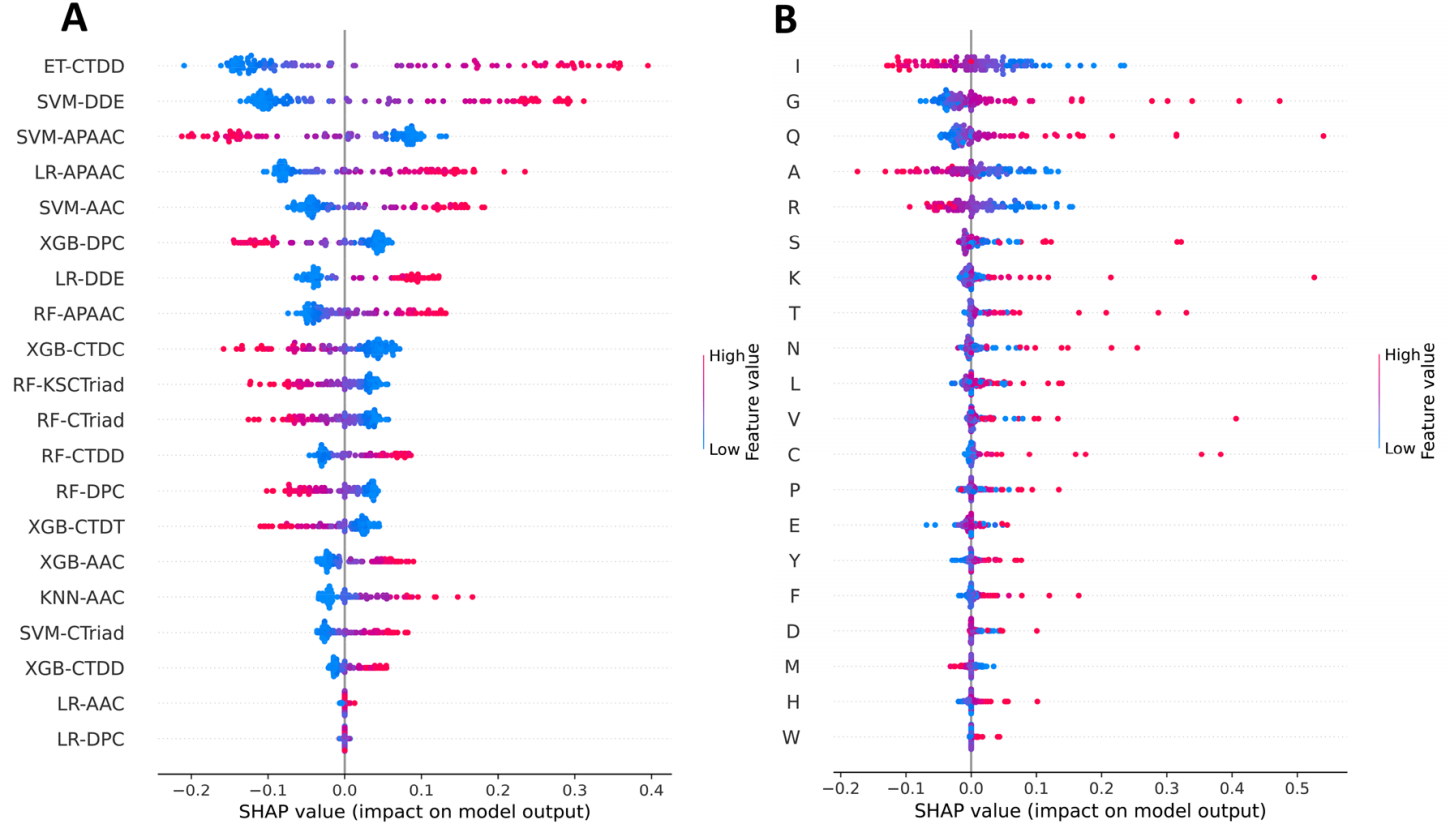
SHAP values of 20 important features used for AMYPred-FRL (**A**) and SVM-AAC (**B**). SHAP values represent the directionality of the informative features, where positive and negative SHAP values represent positive (AMYs) and negative (non-AMYs) predictions.

Here, the SHapley Additive exPlanations (SHAP) approach was utilized to determine which features were the most important for AMYPred-FRL and its constituent baseline models. The SHAP method was well-known as a unified framework that was utilized to enhance interpretable predictions and assess the features’ importance value^53,54^. The AMYPred-FRL was developed by integrating the PFs of the 20 baseline models of SVM-AAC, LR-AAC, XGB-AAC, KNN-AAC, RF-DPC, LR-DPC, XGB-DPC, SVM-APAAC, LR-APAAC, XGB-APAAC, XGB-CTDC, RF-CTDD, ET-CTDD, XGB-CTDD, XGB-CTDT, XGB-KSCTraid, RF-CTraid, XGB-CTraid, SVM-DDE, LR-DDE (Supplementary Table S5). As seen in Fig. 5A, the top five PFs consists of five baseline models of ET-CTDT, SVM-DDE, SVM-APAAC, LR-APAAC and SVM-AAC play an important role for AMYPred-FRL. It could be noticed that SVM-AAC was found in the 5th top-ranked important baseline model ranked by SHAP values. Figure 5B shows that Ile, Gly, Gln, Ala and Arg play a predominant role for SVM-AAC, where Gly and Gln might be crucial factors responsible for AMYs, while Ile, Arg and Ala might be crucial factors responsible for non-AMYs. These results were consistent with the 20 amino acid compositions of AMYs and non-AMYs as summarized in Supplementary Table S6. However, the analysis result was derived from the training dataset containing 132 AMYs and 305 non-AMYs. As a result, this analysis might be limited due to the small size of samples and classes used herein. Improving predictive abilities and model interpretability in future studies will require further computational model development for the AMYs subclass prediction.

### Comparison of AMYPred-FRL and its constituent baseline models

**Figure 6:**
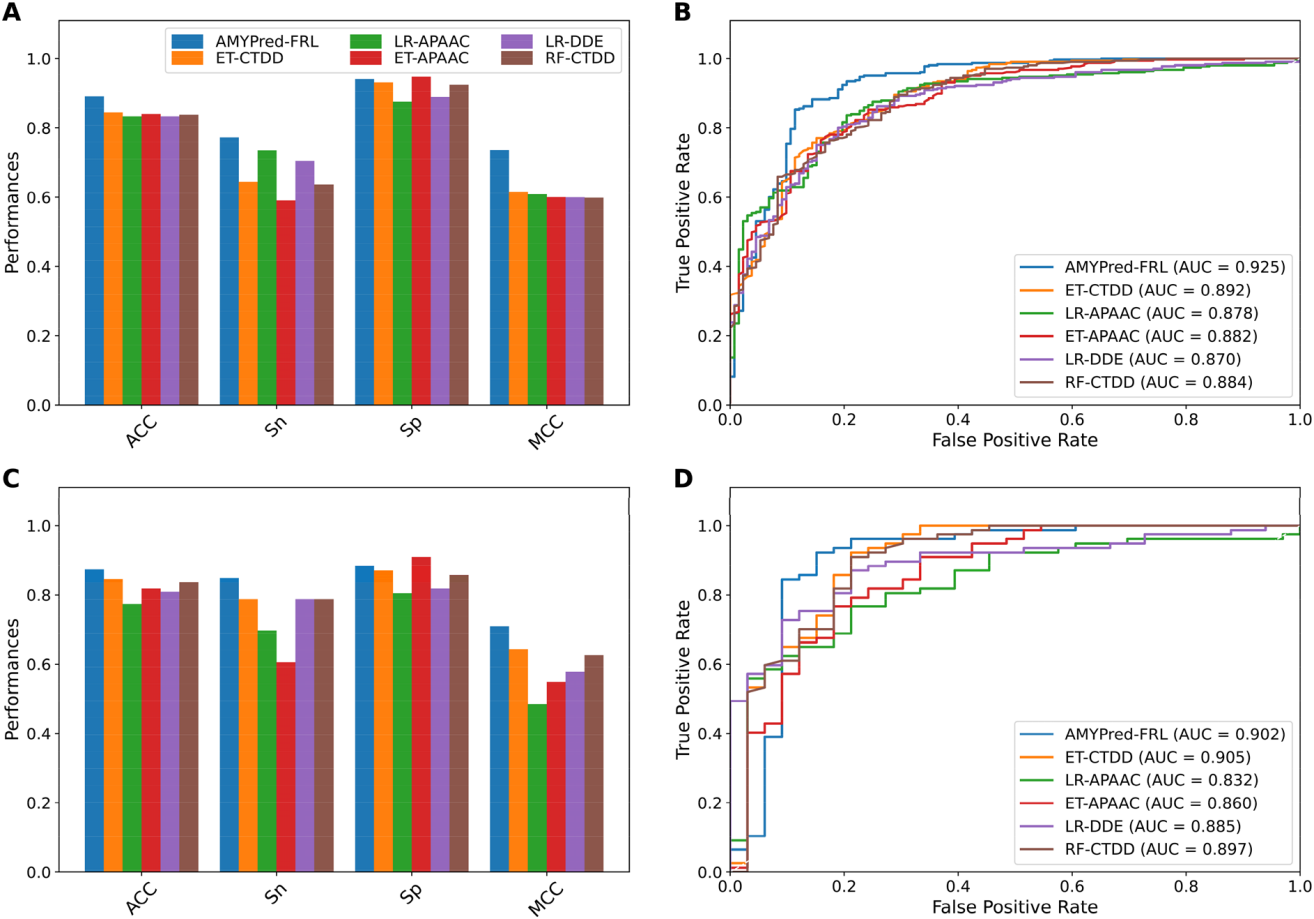
Performance comparison of AMYPred-FRL with the top five baseline models on the training (**A**,**B**) and independent tests (**C**,**D**). Prediction results of AMYPred-FRL and the top five baseline models in terms of ACC, Sn, Sp and MCC (**A**,**C**). ROC curves and AUC values of top five baseline models (**B**,**D**).

To investigate the effectiveness of the AMYPred-FRL predictor, we compared its performance against the top five baseline models having the highest cross-validation ACC and MCC, namely ET-CTDD, LR-APAAC, ET-APAAC, LR-DDE and RF-CTDD,. To create a fair comparison, these top five baseline models were evaluated on the same training and independent datasets. The comparative performance of AMYPred-FRL and the top five baseline models is summarized in Fig. 6. Detailed results are presented in Supplementary Table S7. It can be seen from Fig. 6A,B that AMYPred-FRL afforded the best cross-validation performance as indicated by four out of five evaluation metrics (ACC, Sn, MCC and AUC). In particular, AMYPred-FRL had ACC, Sn, MCC and AUC of 5.0–5.9%, 4.5–14.4%%, 13.3–14.4% and 3.2–5.4%, respectively, higher than the top five baseline models. In the case of models evaluated on the independent test set, AMYPred-FRL was found to produce the best performance as judged by ACC, Sn and MCC (Fig. 6C,D). Notably, the ACC, Sp and MCC of AMYPred-FRL were 0.873, 0.848 and 0.710, respectively, which corresponded to improvements of 1.8–10.0%, 6.0–24.2% and 5.0–22.6% greater than those of the top five baseline models, respectively. In addition, Sp and MCC results from the AMYPred-FRL model demonstrated that it is a powerful AMY predictor that can effectively distinguish false positives and false negatives for unknown AMY candidates, highlighting its superior generalization ability.

### Comparison of AMYPred-FRL with two state-of-the-art methods

To further validate the robustness of AMYPred-FRL, we tested and compared its predictive performance against two of four current state-of-the-art methods (RFAmyloid^20^ and iAMY-SCM^21^) using the independent dataset (33 AMYs and 77 non-AMYs) because the other two state-of-the-art methods (PredAmyl-MLP^22^ and Mukhtar et al.’s method^23^) were not performed using the independent test. Table 6 summarizes the predictive performance of the two compared methods, which were obtained by feeding protein sequences from the independent dataset (i.e. containing 33 AMYs and 77 non-AMYs) to their web servers (accessed on 7 July 2021). As can be seen in Table 6, AMYPred-FRL achieved the best overall performance as indicated by three performance measures (ACC, Sn and MCC) as compared by the two state-of-the-art methods. Particularly, the ACC, Sn and MCC for AMYPred-FRL had corresponding values of 0.873, 0.848, and 0.710, respectively, higher than the second-best method iAMY-SCM by 5.5%, 24.2% and 16.1% respectively. This suggests that the predictor proposed herein was more effective than the compared state-of-the-art methods for distinguishing AMYs from non-AMYs.

**Table 6:** Performance comparison of AMYPred-FRL with the two state-of-the-art methods on as evaluated on the independent test.

Method	ACC	Sn	Sp	MCC
RFAmyloid	0.390	0.656	0.282	-0.061
iAMY-SCM	0.818	0.606	0.909	0.549
AMYPred-FRL	0.873	0.848	0.883	0.710

Performance of RFAmyloid and iAMY-SCM were obtained by feeding protein sequences from the independent dataset to their web servers (accessed on 7 July 2021).

### Case study

In this section, we performed a case study based on an external dataset that was extracted from the CPAD 2.0 database^55^ (downloaded on 16 December 2021) to assess the predictive capability of AMYPred-FRL. We first removed all AMYs and non-AMYs that were found in the training and independent datasets from the Amy dataset^20^. Sequences containing < 20 amino acids were also excluded. As a result, the final external dataset contained 50 AMYs and 19 non-AMYs. Supplementary Tables S8–S10 provides detailed prediction results of AMYPred-FRL, iAMY-SCM and the top three baseline models (i.e., ET-CTDD, LR-APAAC and ET-APAAC) on the external dataset. As seen, AMYPred-FRL achieved the best performance measured by three metrics, including ACC (0.971), Sn (0.980) and MCC (0.928), as compared with iAMY-SCM (Supplementary Table S8) and the best-performing baseline model ET-CTDD (Supplementary Tables S9–S11).

Although ET-CTDD achieved comparable performance with AMYPred-FRL on the external dataset, this method failed to perform well on both the training (Sn of 0.636 and MCC of 0.610) and independent test (Sn of 0.788 and MCC of 0.660) datasets. On the other hand, Supplementary Table S11 shows that the performances of AMYPred-FRL on all the training, independent test and external datasets are consistently better than ET-CTDD and other baseline models. Furthermore, the MCC of AMYPred-FRL on the training and independent test datasets were significantly higher than that of ET-CTDD (0.743 vs. 0.610 and 0.710 vs. 0.660, respectively), highlighting the superior generalization ability of AMYPred-FRL. This indicated that the FRL strategy is capable of effectively integrating the strengths of baseline models to make more accurate and stable AMY identification. And, the high MCC of AMYPred-FRL indicated that this new predictor could effectively reduce the number of both false positive and false negative and narrow down experimental efforts.

### Genome-wide prediction of AMYs in *Saccharomyces cerevisiae*

In this study, we also utilized the proposed AMYPred-FRL for the proteome-wide identification of AMYs for *Saccharomyces cerevisiae.* First of all, we collected 126,486 *Saccharomyces cerevisiae* proteins, which were directly downloaded from the UniProt database. Then, we used the probability thresholds of 0.80, 0.85, 0.90, 0.95 and 0.99 in order to obtain the high-confidence prediction results. The statistical summary of predicted AMYs based on various the probability thresholds are provided in Supplementary Table S12. As seen in Supplementary Table S12, the numbers of predicted AMYs based probability thresholds of 0.80, 0.85, 0.90, 0.95 and 0.99 are 9710, 7028, 4174, 1444 and 105, respectively. Detailed lists of the predicted AMYs based on the five selected probability thresholds could be freely downloaded at http://pmlabstack.pythonanywhere.com/AMYPred-FRL.

## Conclusions

Identification of amyloid proteins is crucial for accelerating the drug development process as well as aiding the understanding of their functional properties. Few computational approaches have been proposed for amyloid protein identification. These models use different approaches to amyloid identification, so could be used together, however there appears to be no computational approach yet developed that can effectively integrate variant models to develop a hybrid model that could achieve high model performance relative to that of the single feature-based approach. Therefore, in this study, we developed AMYPred-FRL as a novel machine-learning meta-predictor for the accurate identification of amyloid proteins by using the FRL approach. Particularly, AMYPred-FRL makes use of ten different feature encodings (AAC, APAAC, CTDC, CTDD, CTDT, CTriad, DPC, DDE, GAAC and KSCTriad) as derived from three different aspects (composition information, composition–transition–distribution information and physicochemical properties) that are subsequently modeled by six well-known ML algorithms (ET, KNN, LR, RF, SVM and XGB). A series of comparative experiments showed that AMYPred-FRL can achieve a better performance than those of its constituent baseline models and state-of-the-art methods (RFAmyloid and iAMY-SCM) as evaluated on the independent test thereby highlighting its effectiveness and generalization ability. A user-friendly web server of AMYPred-FRL is freely available at http://pmlabstack.pythonanywhere.com/AMYPred-FRL. It is anticipated that the proposed AMYPred-FRL would enable biologists to rapidly identify amyloid proteins.

## Supplementary Information

Supplementary Tables.

## Data Availability

All the data used in this study are available at http://pmlabstack.pythonanywhere.com/AMYPred-FRL.
