# Supplementary material for: AMYPred-FRL is a novel approach for accurate prediction of amyloid proteins by using feature representation learning
Source: Sci Rep. 2022 May 11;12:7697. doi: 10.1038/s41598-022-11897-z (PMC9095707; doi:10.1038/s41598-022-11897-z)
Supplement: Supplementary file 1 — Supplementary Tables. [file 41598_2022_11897_MOESM1_ESM.docx]

**Table S1.** The groups of GAAC.

| Physiochemical Properties Amino Acid |
| --- |
| Aliphatic G,A,V,L,M,I |
| Aromatic F,Y,W |
| Positive Charge K,R,H |
| Negative Charge D,E |
| Uncharged S,T,P,C,N,Q |

**Table S2.** Hyperparameter search details for six different ML classifiers.

| **Method** | **Parameters** | **Range of parameters** |
| --- | --- | --- |
| ET | n_estimators | [20, 50, 100, 200, 500] |
| KNN | K values | Default |
| LR | C | [0.001, 0.01, 0.1, 1, 10, 100] |
| RF | n_estimators | [20, 50, 100, 200, 500] |
| SVM | C | [1, 2, 4, 8, 16, 32] |
| XGB | n_estimators | [50, 100, 200, 500] |

Columns 2 and 3 represents the parameter name used in the Scikit-learn library and the range of parameter used to develop the model, respectively.

**Table S3** Cross-validation results of 60 baseline models developed using six different ML algorithms and ten feature descriptors.

| **Descriptor** | **Method** | **ACC** | **Sn** | **Sp** | **MCC** | **AUC** |
| --- | --- | --- | --- | --- | --- | --- |
| AAC | ET | 0.810 | 0.470 | 0.957 | 0.520 | 0.851 |
|  | KNN | 0.757 | 0.288 | 0.961 | 0.358 | 0.773 |
|  | LR | 0.698 | 0.000 | 1.000 | 0.000 | 0.683 |
|  | RF | 0.794 | 0.500 | 0.921 | 0.478 | 0.829 |
|  | SVM | 0.810 | 0.553 | 0.921 | 0.524 | 0.850 |
|  | XGB | 0.801 | 0.500 | 0.931 | 0.496 | 0.810 |
| APAAC | ET | 0.840 | 0.591 | 0.948 | 0.602 | 0.882 |
|  | KNN | 0.812 | 0.591 | 0.908 | 0.535 | 0.848 |
|  | LR | 0.833 | 0.735 | 0.875 | 0.606 | 0.878 |
|  | RF | 0.815 | 0.545 | 0.931 | 0.535 | 0.873 |
|  | SVM | 0.801 | 0.621 | 0.879 | 0.516 | 0.828 |
|  | XGB | 0.833 | 0.644 | 0.915 | 0.589 | 0.881 |
| CTDC | ET | 0.755 | 0.371 | 0.921 | 0.360 | 0.778 |
|  | KNN | 0.735 | 0.242 | 0.948 | 0.279 | 0.678 |
|  | LR | 0.705 | 0.045 | 0.990 | 0.115 | 0.636 |
|  | RF | 0.744 | 0.394 | 0.895 | 0.337 | 0.767 |
|  | SVM | 0.746 | 0.280 | 0.948 | 0.320 | 0.733 |
|  | XGB | 0.721 | 0.356 | 0.879 | 0.274 | 0.744 |
| CTDD | ET | 0.842 | 0.636 | 0.931 | 0.610 | 0.892 |
|  | KNN | 0.780 | 0.333 | 0.974 | 0.436 | 0.752 |
|  | LR | 0.794 | 0.636 | 0.862 | 0.505 | 0.782 |
|  | RF | 0.838 | 0.636 | 0.925 | 0.599 | 0.884 |
|  | SVM | 0.776 | 0.386 | 0.944 | 0.419 | 0.812 |
|  | XGB | 0.817 | 0.583 | 0.918 | 0.544 | 0.866 |
| CTDT | ET | 0.776 | 0.424 | 0.928 | 0.422 | 0.805 |
|  | KNN | 0.728 | 0.197 | 0.957 | 0.249 | 0.679 |
|  | LR | 0.698 | 0.000 | 1.000 | 0.000 | 0.602 |
|  | RF | 0.748 | 0.424 | 0.889 | 0.355 | 0.793 |
|  | SVM | 0.776 | 0.409 | 0.934 | 0.421 | 0.774 |
|  | XGB | 0.746 | 0.439 | 0.879 | 0.354 | 0.761 |
| CTraid | ET | 0.799 | 0.477 | 0.938 | 0.488 | 0.862 |
|  | KNN | 0.741 | 0.205 | 0.974 | 0.302 | 0.727 |
|  | LR | 0.778 | 0.485 | 0.905 | 0.437 | 0.789 |
|  | RF | 0.808 | 0.553 | 0.918 | 0.519 | 0.847 |
|  | SVM | 0.801 | 0.583 | 0.895 | 0.508 | 0.840 |
|  | XGB | 0.792 | 0.530 | 0.905 | 0.477 | 0.834 |
| DDE | ET | 0.812 | 0.508 | 0.944 | 0.526 | 0.864 |
|  | KNN | 0.803 | 0.652 | 0.869 | 0.527 | 0.827 |
|  | LR | 0.833 | 0.705 | 0.889 | 0.600 | 0.870 |
|  | RF | 0.801 | 0.508 | 0.928 | 0.496 | 0.851 |
|  | SVM | 0.835 | 0.659 | 0.911 | 0.597 | 0.890 |
|  | XGB | 0.810 | 0.568 | 0.915 | 0.526 | 0.862 |
| DPC | ET | 0.808 | 0.485 | 0.948 | 0.513 | 0.864 |
|  | KNN | 0.723 | 0.098 | 0.993 | 0.232 | 0.625 |
|  | LR | 0.698 | 0.000 | 1.000 | 0.000 | 0.662 |
|  | RF | 0.801 | 0.568 | 0.902 | 0.505 | 0.857 |
|  | SVM | 0.817 | 0.591 | 0.915 | 0.545 | 0.862 |
|  | XGB | 0.792 | 0.568 | 0.889 | 0.485 | 0.859 |
| GAAC | ET | 0.714 | 0.364 | 0.866 | 0.261 | 0.676 |
|  | KNN | 0.714 | 0.341 | 0.875 | 0.253 | 0.634 |
|  | LR | 0.698 | 0.000 | 1.000 | 0.000 | 0.616 |
|  | RF | 0.719 | 0.371 | 0.869 | 0.274 | 0.690 |
|  | SVM | 0.723 | 0.182 | 0.957 | 0.230 | 0.653 |
|  | XGB | 0.707 | 0.341 | 0.866 | 0.238 | 0.660 |
| KSCTriad | ET | 0.801 | 0.477 | 0.941 | 0.494 | 0.857 |
|  | KNN | 0.773 | 0.303 | 0.977 | 0.415 | 0.733 |
|  | LR | 0.741 | 0.447 | 0.869 | 0.346 | 0.758 |
|  | RF | 0.810 | 0.538 | 0.928 | 0.522 | 0.840 |
|  | SVM | 0.789 | 0.538 | 0.898 | 0.474 | 0.826 |
|  | XGB | 0.805 | 0.545 | 0.918 | 0.512 | 0.862 |

**Table S4** Independent test results of 60 baseline models developed using six different ML algorithms and ten feature descriptors.

| **Descriptor** | **Method** | **ACC** | **Sn** | **Sp** | **MCC** | **AUC** |
| --- | --- | --- | --- | --- | --- | --- |
| AAC | ET | 0.836 | 0.606 | 0.935 | 0.592 | 0.850 |
|  | KNN | 0.773 | 0.303 | 0.974 | 0.407 | 0.763 |
|  | LR | 0.700 | 0.000 | 1.000 | 0.000 | 0.705 |
|  | RF | 0.845 | 0.636 | 0.935 | 0.616 | 0.853 |
|  | SVM | 0.809 | 0.636 | 0.883 | 0.535 | 0.852 |
|  | XGB | 0.845 | 0.697 | 0.909 | 0.624 | 0.848 |
| APAAC | ET | 0.818 | 0.606 | 0.909 | 0.549 | 0.860 |
|  | KNN | 0.764 | 0.667 | 0.805 | 0.458 | 0.829 |
|  | LR | 0.773 | 0.697 | 0.805 | 0.484 | 0.832 |
|  | RF | 0.809 | 0.576 | 0.909 | 0.523 | 0.852 |
|  | SVM | 0.755 | 0.606 | 0.818 | 0.421 | 0.820 |
|  | XGB | 0.809 | 0.667 | 0.870 | 0.542 | 0.876 |
| CTDC | ET | 0.782 | 0.455 | 0.922 | 0.439 | 0.806 |
|  | KNN | 0.727 | 0.242 | 0.935 | 0.252 | 0.747 |
|  | LR | 0.709 | 0.061 | 0.987 | 0.134 | 0.636 |
|  | RF | 0.773 | 0.455 | 0.909 | 0.417 | 0.778 |
|  | SVM | 0.727 | 0.303 | 0.909 | 0.269 | 0.722 |
|  | XGB | 0.709 | 0.394 | 0.844 | 0.260 | 0.763 |
| CTDD | ET | 0.855 | 0.788 | 0.883 | 0.660 | 0.905 |
|  | KNN | 0.800 | 0.424 | 0.961 | 0.488 | 0.723 |
|  | LR | 0.782 | 0.697 | 0.818 | 0.500 | 0.764 |
|  | RF | 0.836 | 0.788 | 0.857 | 0.626 | 0.897 |
|  | SVM | 0.791 | 0.455 | 0.935 | 0.463 | 0.769 |
|  | XGB | 0.873 | 0.758 | 0.922 | 0.692 | 0.878 |
| CTDT | ET | 0.809 | 0.515 | 0.935 | 0.516 | 0.835 |
|  | KNN | 0.727 | 0.182 | 0.961 | 0.239 | 0.712 |
|  | LR | 0.709 | 0.030 | 1.000 | 0.146 | 0.710 |
|  | RF | 0.800 | 0.606 | 0.883 | 0.509 | 0.827 |
|  | SVM | 0.791 | 0.485 | 0.922 | 0.466 | 0.809 |
|  | XGB | 0.782 | 0.545 | 0.883 | 0.456 | 0.832 |
| CTraid | ET | 0.773 | 0.545 | 0.870 | 0.437 | 0.850 |
|  | KNN | 0.764 | 0.212 | 1.000 | 0.398 | 0.784 |
|  | LR | 0.736 | 0.485 | 0.844 | 0.346 | 0.773 |
|  | RF | 0.773 | 0.576 | 0.857 | 0.445 | 0.834 |
|  | SVM | 0.755 | 0.545 | 0.844 | 0.401 | 0.801 |
|  | XGB | 0.800 | 0.576 | 0.896 | 0.502 | 0.839 |
| DDE | ET | 0.800 | 0.545 | 0.909 | 0.497 | 0.866 |
|  | KNN | 0.827 | 0.788 | 0.844 | 0.609 | 0.846 |
|  | LR | 0.809 | 0.788 | 0.818 | 0.577 | 0.885 |
|  | RF | 0.782 | 0.576 | 0.870 | 0.464 | 0.866 |
|  | SVM | 0.791 | 0.667 | 0.844 | 0.507 | 0.867 |
|  | XGB | 0.791 | 0.606 | 0.870 | 0.490 | 0.890 |
| DPC | ET | 0.791 | 0.515 | 0.909 | 0.471 | 0.857 |
|  | KNN | 0.718 | 0.061 | 1.000 | 0.208 | 0.654 |
|  | LR | 0.700 | 0.000 | 1.000 | 0.000 | 0.695 |
|  | RF | 0.791 | 0.606 | 0.870 | 0.490 | 0.854 |
|  | SVM | 0.782 | 0.636 | 0.844 | 0.481 | 0.861 |
|  | XGB | 0.773 | 0.576 | 0.857 | 0.445 | 0.860 |
| GAAC | ET | 0.655 | 0.273 | 0.818 | 0.102 | 0.728 |
|  | KNN | 0.700 | 0.303 | 0.870 | 0.206 | 0.737 |
|  | LR | 0.700 | 0.000 | 1.000 | 0.000 | 0.619 |
|  | RF | 0.664 | 0.303 | 0.818 | 0.134 | 0.695 |
|  | SVM | 0.727 | 0.242 | 0.935 | 0.252 | 0.708 |
|  | XGB | 0.682 | 0.333 | 0.831 | 0.183 | 0.685 |
| KSCTriad | ET | 0.800 | 0.545 | 0.909 | 0.497 | 0.860 |
|  | KNN | 0.791 | 0.333 | 0.987 | 0.471 | 0.759 |
|  | LR | 0.736 | 0.424 | 0.870 | 0.327 | 0.704 |
|  | RF | 0.764 | 0.576 | 0.844 | 0.428 | 0.851 |
|  | SVM | 0.791 | 0.545 | 0.896 | 0.476 | 0.825 |
|  | XGB | 0.745 | 0.515 | 0.844 | 0.374 | 0.819 |

**Table S5** Details of the optimal feature vectors of CF, PF and PCF.

| **Feature** | **#Feature** | **Detail** |
| --- | --- | --- |
| Optimal CF | 30 | SVM-AAC, SVM-DPC, SVM-APAAC, SVM-CTDC, SVM-CTDD, SVM-GAAC, SVM-CTriad, SVM-DDE, RF-CTDC, RF-CTDD, RF-CTDT, RF-GAAC, ET-CTDD, ET-CTDT, ET-KSCTriad, ET-CTriad, XGB-AAC, XGB-CTDC, XGB-GAAC, XGB-CTriad, XGB-DDE, KNN-CTDC, KNN-KSCTriad, KNN-DDE, LR-AAC, LR-APAAC, LR-CTDC, LR-CTDD, LR-GAAC, LR-KSCTriad |
| Optimal PF | 20 | SVM-AAC, LR-AAC, XGB-AAC, KNN-AAC, RF-DPC, LR-DPC, XGB-DPC, SVM-APAAC, LR-APAAC, XGB-APAAC, XGB-CTDC, RF-CTDD, ET-CTDD, XGB-CTDD, XGB-CTDT, XGB-KSCTraid, RF-CTraid, XGB-CTraid, SVM-DDE, LR-DDE |
| Optimal PCF | 10 | SVM-CTDD-PF, RF-AAC-PF, RF-CTDT-PF, ET-CTDD-PF, ET-CTDT-PF, LR-CTDC-PF, ET-GAAC-CF, XGB-AAC-CF, KNN-KSCTriad-CF, LR-KSCTriad-CF |

**Table S6.** Amino acid compositions (%) of amyloid (AMY) and non-amyloid (Non-AMY) proteins.

| Amino acid | AMY (%) | Non-AMY (%) | Difference (Rank) |
| --- | --- | --- | --- |
| Q-Gln | 5.45 | 4.17 | 1.28(1) |
| K-Lys | 5.80 | 5.28 | 0.52(5) |
| C-Cys | 1.93 | 1.50 | 0.43(6) |
| T-Thr | 5.63 | 5.60 | 0.03(9) |
| W-Trp | 1.18 | 1.26 | -0.07(11) |
| H-His | 2.19 | 2.23 | -0.04(10) |
| S-Ser | 8.35 | 7.58 | 0.77(4) |
| G-Gly | 7.96 | 6.77 | 1.19(2) |
| E-Glu | 5.99 | 6.38 | -0.39(15) |
| P-Pro | 4.53 | 5.17 | -0.65(16) |
| N-Asn | 5.42 | 4.32 | 1.10(3) |
| Y-Tyr | 3.19 | 2.99 | 0.20(8) |
| M-Met | 2.12 | 2.19 | -0.07(12) |
| F-Phe | 4.06 | 3.76 | 0.31(7) |
| V-Val | 6.41 | 6.54 | -0.13(13) |
| D-Asp | 5.00 | 5.36 | -0.36(14) |
| A-Ala | 7.57 | 8.64 | -1.07(19) |
| L-Leu | 8.35 | 9.24 | -0.89(18) |
| R-Arg | 4.31 | 5.73 | -1.43(20) |
| I-Ile | 4.55 | 5.28 | -0.73(17) |

**Table S7** Performance comparison of AMYPred-FRL and the top-five baseline models on the training and independent datasets.

| **Dataset** | **Model** | **ACC** | **Sn** | **Sp** | **MCC** | **AUC** |
| --- | --- | --- | --- | --- | --- | --- |
| Training set | AMYPred-FRL | 0.892 | 0.780 | 0.941 | 0.743 | 0.925 |
|  | ET-CTDD | 0.842 | 0.636 | 0.931 | 0.610 | 0.892 |
|  | LR-APAAC | 0.833 | 0.735 | 0.875 | 0.606 | 0.878 |
|  | ET-APAAC | 0.840 | 0.591 | 0.948 | 0.602 | 0.882 |
|  | LR-DDE | 0.833 | 0.705 | 0.889 | 0.600 | 0.870 |
|  | RF-CTDD | 0.838 | 0.636 | 0.925 | 0.599 | 0.884 |
| Independent set | AMYPred-FRL | 0.873 | 0.848 | 0.883 | 0.710 | 0.902 |
|  | ET-CTDD | 0.855 | 0.788 | 0.883 | 0.660 | 0.905 |
|  | LR-APAAC | 0.773 | 0.697 | 0.805 | 0.484 | 0.832 |
|  | ET-APAAC | 0.818 | 0.606 | 0.909 | 0.549 | 0.860 |
|  | LR-DDE | 0.809 | 0.788 | 0.818 | 0.577 | 0.885 |
|  | RF-CTDD | 0.836 | 0.788 | 0.857 | 0.626 | 0.897 |

**Table S8** Detailed prediction results of AMYPred-FRL and iAMY-SCM on the external dataset.

| **#** | **Sequence name** | **TRUE** | **iAMY-SCM** | | **AMYPred-FRL** | |
| --- | --- | --- | --- | --- | --- | --- |
|  |  |  | **Score** | **Prediction** | **Score** | **Prediction** |
| 1 | P-1873 | AMY | 296.74 | AMY | 0.93 | AMY |
| 2 | P-1875 | AMY | 256.05 | non-AMY | 0.9 | AMY |
| 3 | P-1876 | AMY | 212.74 | non-AMY | 0.4 | non-AMY |
| 4 | P-1879 | AMY | 217.26 | non-AMY | 0.92 | AMY |
| 5 | P-1882 | AMY | 265.89 | non-AMY | 0.89 | AMY |
| 6 | P-1883 | AMY | 281.68 | non-AMY | 0.91 | AMY |
| 7 | P-1885 | AMY | 266.45 | non-AMY | 0.94 | AMY |
| 8 | P-1888 | AMY | 338.8 | AMY | 0.72 | AMY |
| 9 | P-1890 | AMY | 216.65 | non-AMY | 0.95 | AMY |
| 10 | P-1896 | AMY | 299.3 | AMY | 0.89 | AMY |
| 11 | P-1897 | AMY | 332.5 | AMY | 0.89 | AMY |
| 12 | P-1899 | AMY | 439.48 | AMY | 0.85 | AMY |
| 13 | P-1900 | AMY | 259.9 | non-AMY | 0.78 | AMY |
| 14 | P-1903 | AMY | 363.19 | AMY | 0.91 | AMY |
| 15 | P-1917 | AMY | 302.18 | AMY | 0.91 | AMY |
| 16 | P-1920 | AMY | 274.96 | non-AMY | 0.89 | AMY |
| 17 | P-1924 | AMY | 341.65 | AMY | 0.94 | AMY |
| 18 | P-1925 | AMY | 411.58 | AMY | 0.86 | AMY |
| 19 | P-1926 | AMY | 401.88 | AMY | 0.85 | AMY |
| 20 | P-1928 | AMY | 294.42 | AMY | 0.93 | AMY |
| 21 | P-1930 | AMY | 292.96 | AMY | 0.94 | AMY |
| 22 | P-1932 | AMY | 252.16 | non-AMY | 0.87 | AMY |
| 23 | P-1933 | AMY | 258.92 | non-AMY | 0.92 | AMY |
| 24 | P-1938 | AMY | 295.12 | AMY | 0.93 | AMY |
| 25 | P-1939 | AMY | 299.89 | AMY | 0.9 | AMY |
| 26 | P-1942 | AMY | 321.93 | AMY | 0.96 | AMY |
| 27 | P-1943 | AMY | 207.89 | non-AMY | 0.77 | AMY |
| 28 | P-1944 | AMY | 207.48 | non-AMY | 0.63 | AMY |
| 29 | P-1945 | AMY | 208 | non-AMY | 0.53 | AMY |
| 30 | P-1946 | AMY | 210.93 | non-AMY | 0.61 | AMY |
| 31 | P-1947 | AMY | 219.15 | non-AMY | 0.78 | AMY |
| 32 | P-1948 | AMY | 205.74 | non-AMY | 0.84 | AMY |
| 33 | P-1950 | AMY | 209.82 | non-AMY | 0.58 | AMY |
| 34 | P-1951 | AMY | 358.79 | AMY | 0.93 | AMY |
| 35 | P-1952 | AMY | 366.68 | AMY | 0.92 | AMY |
| 36 | P-1953 | AMY | 337.68 | AMY | 0.93 | AMY |
| 37 | P-1954 | AMY | 333.54 | AMY | 0.92 | AMY |
| 38 | P-1959 | AMY | 273.83 | non-AMY | 0.95 | AMY |
| 39 | P-1970 | AMY | 268.76 | non-AMY | 0.76 | AMY |
| 40 | P-1980 | AMY | 289.31 | AMY | 0.95 | AMY |
| 41 | P-2003 | AMY | 290.56 | AMY | 0.97 | AMY |
| 42 | P-2006 | AMY | 292.82 | AMY | 0.93 | AMY |
| 43 | P-2009 | AMY | 277.39 | non-AMY | 0.95 | AMY |
| 44 | P-2011 | AMY | 285.2 | non-AMY | 0.93 | AMY |
| 45 | P-2019 | AMY | 354.76 | AMY | 0.92 | AMY |
| 46 | P-2020 | AMY | 274.67 | non-AMY | 0.93 | AMY |
| 47 | P-2021 | AMY | 267.51 | non-AMY | 0.93 | AMY |
| 48 | P-2022 | AMY | 660.23 | AMY | 0.59 | AMY |
| 49 | P-2024 | AMY | 220.44 | non-AMY | 0.73 | AMY |
| 50 | P-2029 | AMY | 527.21 | AMY | 0.51 | AMY |
| 51 | P-1866 | non-AMY | 302.11 | AMY | 0.08 | non-AMY |
| 52 | P-1867 | non-AMY | 298.58 | AMY | 0.09 | non-AMY |
| 53 | P-1870 | non-AMY | 271.89 | non-AMY | 0.1 | non-AMY |
| 54 | P-1872 | non-AMY | 324.58 | AMY | 0.07 | non-AMY |
| 55 | P-1880 | non-AMY | 219.21 | non-AMY | 0.09 | non-AMY |
| 56 | P-1881 | non-AMY | 334.47 | AMY | 0.05 | non-AMY |
| 57 | P-1895 | non-AMY | 259.25 | non-AMY | 0.04 | non-AMY |
| 58 | P-1912 | non-AMY | 337.71 | AMY | 0.18 | non-AMY |
| 59 | P-1913 | non-AMY | 385.76 | AMY | 0.08 | non-AMY |
| 60 | P-1915 | non-AMY | 342.23 | AMY | 0.09 | non-AMY |
| 61 | P-1916 | non-AMY | 281.36 | non-AMY | 0.08 | non-AMY |
| 62 | P-1923 | non-AMY | 324.43 | AMY | 0.08 | non-AMY |
| 63 | P-1931 | non-AMY | 308.79 | AMY | 0.19 | non-AMY |
| 64 | P-1937 | non-AMY | 315.32 | AMY | 0.41 | non-AMY |
| 65 | P-1941 | non-AMY | 253.81 | non-AMY | 0.13 | non-AMY |
| 66 | P-1958 | non-AMY | 314.41 | AMY | 0.04 | non-AMY |
| 67 | P-1972 | non-AMY | 328.65 | AMY | 0.07 | non-AMY |
| 68 | P-1976 | non-AMY | 333.11 | AMY | 0.27 | non-AMY |
| 69 | P-1977 | non-AMY | 314.71 | AMY | 0.94 | AMY |

^a^ iAMY-SCM with a threshold of 288.56 was used to identify AMYs

^b^ AMYPred-FRL with default parameters (threshold of 0.5) was used to identify AMYs

**Table S9** Detailed prediction results of AMYPred-FRL and top three baseline models on the external dataset.

| **Sequence Name** | **TRUE** | **AMYPred-FRL** | **ET-CTDD** | **LR-APAAC** | **ET-APAAC** |
| --- | --- | --- | --- | --- | --- |
| P-1873 | AMY | AMY | AMY | AMY | AMY |
| P-1875 | AMY | AMY | AMY | AMY | AMY |
| P-1876 | AMY | non-AMY | AMY | AMY | AMY |
| P-1879 | AMY | AMY | AMY | AMY | AMY |
| P-1882 | AMY | AMY | AMY | AMY | AMY |
| P-1883 | AMY | AMY | AMY | AMY | AMY |
| P-1885 | AMY | AMY | AMY | AMY | AMY |
| P-1888 | AMY | AMY | AMY | AMY | AMY |
| P-1890 | AMY | AMY | AMY | AMY | AMY |
| P-1896 | AMY | AMY | AMY | AMY | AMY |
| P-1897 | AMY | AMY | AMY | AMY | AMY |
| P-1899 | AMY | AMY | AMY | AMY | AMY |
| P-1900 | AMY | AMY | AMY | AMY | AMY |
| P-1903 | AMY | AMY | AMY | AMY | AMY |
| P-1917 | AMY | AMY | AMY | AMY | AMY |
| P-1920 | AMY | AMY | AMY | AMY | AMY |
| P-1924 | AMY | AMY | AMY | AMY | AMY |
| P-1925 | AMY | AMY | AMY | AMY | AMY |
| P-1926 | AMY | AMY | AMY | AMY | AMY |
| P-1928 | AMY | AMY | AMY | AMY | AMY |
| P-1930 | AMY | AMY | AMY | AMY | AMY |
| P-1932 | AMY | AMY | AMY | AMY | AMY |
| P-1933 | AMY | AMY | AMY | AMY | AMY |
| P-1938 | AMY | AMY | AMY | AMY | AMY |
| P-1939 | AMY | AMY | AMY | AMY | AMY |
| P-1942 | AMY | AMY | AMY | AMY | AMY |
| P-1943 | AMY | AMY | AMY | AMY | AMY |
| P-1944 | AMY | AMY | AMY | AMY | AMY |
| P-1945 | AMY | AMY | AMY | AMY | AMY |
| P-1946 | AMY | AMY | AMY | AMY | AMY |
| P-1947 | AMY | AMY | AMY | AMY | AMY |
| P-1948 | AMY | AMY | AMY | AMY | AMY |
| P-1950 | AMY | AMY | AMY | AMY | AMY |
| P-1951 | AMY | AMY | AMY | AMY | AMY |
| P-1952 | AMY | AMY | AMY | AMY | AMY |
| P-1953 | AMY | AMY | AMY | AMY | AMY |
| P-1954 | AMY | AMY | AMY | AMY | AMY |
| P-1959 | AMY | AMY | AMY | AMY | AMY |
| P-1970 | AMY | AMY | AMY | AMY | AMY |
| P-1980 | AMY | AMY | AMY | AMY | AMY |
| P-2003 | AMY | AMY | AMY | AMY | AMY |
| P-2006 | AMY | AMY | AMY | AMY | AMY |
| P-2009 | AMY | AMY | AMY | AMY | AMY |
| P-2011 | AMY | AMY | AMY | AMY | AMY |
| P-2019 | AMY | AMY | AMY | AMY | AMY |
| P-2020 | AMY | AMY | AMY | AMY | AMY |
| P-2021 | AMY | AMY | AMY | AMY | AMY |
| P-2022 | AMY | AMY | AMY | AMY | AMY |
| P-2024 | AMY | AMY | AMY | AMY | AMY |
| P-2029 | AMY | AMY | AMY | AMY | AMY |
| P-1 | non-AMY | non-AMY | non-AMY | non-AMY | non-AMY |
| P-2 | non-AMY | non-AMY | non-AMY | non-AMY | non-AMY |
| P-3 | non-AMY | non-AMY | non-AMY | non-AMY | non-AMY |
| P-4 | non-AMY | non-AMY | non-AMY | non-AMY | non-AMY |
| P-5 | non-AMY | non-AMY | non-AMY | non-AMY | non-AMY |
| P-6 | non-AMY | non-AMY | non-AMY | non-AMY | non-AMY |
| P-7 | non-AMY | non-AMY | non-AMY | non-AMY | non-AMY |
| P-8 | non-AMY | non-AMY | non-AMY | non-AMY | non-AMY |
| P-9 | non-AMY | non-AMY | non-AMY | non-AMY | non-AMY |
| P-10 | non-AMY | non-AMY | non-AMY | non-AMY | non-AMY |
| P-11 | non-AMY | non-AMY | non-AMY | non-AMY | non-AMY |
| P-12 | non-AMY | non-AMY | non-AMY | non-AMY | non-AMY |
| P-13 | non-AMY | non-AMY | non-AMY | non-AMY | non-AMY |
| P-14 | non-AMY | non-AMY | AMY | AMY | non-AMY |
| P-15 | non-AMY | non-AMY | non-AMY | AMY | non-AMY |
| P-16 | non-AMY | non-AMY | non-AMY | non-AMY | non-AMY |
| P-17 | non-AMY | non-AMY | non-AMY | non-AMY | non-AMY |
| P-18 | non-AMY | non-AMY | AMY | AMY | AMY |
| P-19 | non-AMY | AMY | AMY | AMY | AMY |

**Table S10** Performance comparison of AMYPred-FRL and top three baseline models on the external dataset.

| Model | ACC | Sn | Sp | MCC |
| --- | --- | --- | --- | --- |
| AMYPred-FRL | 0.971 | 0.980 | 0.947 | 0.927 |
| ET-CTDD | 0.957 | 0.943 | 1.000 | 0.891 |
| LR-APAAC | 0.942 | 0.926 | 1.000 | 0.855 |
| ET-APAAC | 0.971 | 0.962 | 1.000 | 0.928 |

**Table S11** Performance comparison of AMYPred-FRL and the best-performing baseline model on the training, independent test and external datasets.

| **Dataset** | **Model** | **ACC** | **Sn** | **Sp** | **MCC** |
| --- | --- | --- | --- | --- | --- |
| Training set | AMYPred-FRL | 0.892 | 0.780 | 0.941 | 0.743 |
|  | ET-CTDD | 0.842 | 0.636 | 0.931 | 0.610 |
| Independent set | AMYPred-FRL | 0.873 | 0.848 | 0.883 | 0.710 |
|  | ET-CTDD | 0.855 | 0.788 | 0.883 | 0.660 |
| External set | AMYPred-FRL | 0.971 | 0.980 | 0.947 | 0.927 |
|  | ET-CTDD | 0.957 | 0.943 | 1.000 | 0.891 |

**Table S12** Numbers of predicted AMYs based the probability thresholds of 0.80, 0.85, 0.90, 0.95 and 0.99.

| **Probability threshold** | **Numbers of predicted AMYs** |
| --- | --- |
| 0.80 | 9,710 |
| 0.85 | 7,028 |
| 0.90 | 4,174 |
| 0.95 | 1,444 |
| 0.99 | 105 |
